# Telocytes in human heart valves

**DOI:** 10.1111/jcmm.12285

**Published:** 2014-03-26

**Authors:** Yang Yang, Wei Sun, Sean M Wu, Junjie Xiao, Xiangqing Kong

**Affiliations:** a Department of Cardiology, The First Affiliated Hospital of Nanjing Medical UniversityNanjing, China; b Cardiovascular Institute, Institute for Stem Cell Biology and regenerative Medicine, Stanford University of MedicineStanford, CA, USA; c Regeneration Lab, School of Life Science, Shanghai UniversityShanghai, China

**Keywords:** telocytes, telopodes, heart valves, CD34, C-kit, vimentin, PDGF-β

## Abstract

Valve interstitial cells (VICs) are responsible for maintaining the structural integrity and dynamic behaviour of the valve. Telocytes (TCs), a peculiar type of interstitial cells, have been recently identified by Popescu*s group in epicardium, myocardium and endocardium (visit http://www.telocytes.com). The presence of TCs has been identified in atria, ventricles and many other tissues and organ, but not yet in heart valves. We used transmission electron microscopy and immunofluorescence methods (double labelling for CD34 and c-kit, or vimentin, or PDGF Receptor-β) to provide evidence for the existence of TCs in human heart valves, including mitral valve, tricuspid valve and aortic valve. TCs are found in both apex and base of heart valves, with a similar density of 27–28 cells/mm^2^ in mitral valve, tricuspid valve and aortic valve. Since TCs are known for the participation in regeneration or repair biological processes, it remains to be determined how TCs contributes to the valve attempts to re-establish normal structure and function following injury, especially a complex junction was found between TCs and a putative stem (progenitor) cell.

## Introduction

Heart valves are outgrowths from the endocardium which enable the blood to flow in a unidirectional way [1]. The heart valve is an intimal sheet mainly with a core containing loose connective tissue near the surface of the atrioventricular orifice and a thick, dense connective tissue plate on the opposite side [1–4]. Valves abnormality will either lead to congenital valve defects or adult valvular heart diseases [1–4]. Any further deep understanding about the histology of heart valves will continue to suggest new therapeutic modalities [1–4].

Two types of cells have been identified within heart valves including endothelial cells and valve interstitial cells (VICs) [5]. Endothelial cells cover the surface of the cusps while VICs form a network within the extracellular matrix in the body of the cusp [5]. Valve interstitial cells are the most abundant cell types in heart valves and are responsible for the secretion of valve matrix such as collagen, proteoglycans and elastin [6–8]. Thus, VICs participate in the pathological processes associated with collagen fibre disorganization, leaflet thickening and calcification [6,9].

Telocytes (TCs), a special type of interstitial cells, have been identified by Popescu*s group [10–27] and the concept of TCs has been adopted by many laboratories worldwide [28–42] (for details visit http://www.telocytes.com). Telocytes are described as interstitial cells with extremely long and thin prolongations, called telopodes (Tps) [10,11,18]. The length of Tps can be from several tens to hundreds of micrometres, forming junctions with a variety of cells [10,11,18]. Actually, TCs make a 3D network in the heart [10,14]. Tp have dilated portions (podoms) and very thin segments (podomeres), usually below the resolving power of light microscopy [10]. This explains the fact that TCs were overlooked, as an interstitial cell type. Telocytes are not excitable cells [11,13]. They release ectosomes, usually as cangos [18,19]. The microRNA signatures as well as the gene profiles were described [25,43]. Moreover, TCs with similar ultrastructural and phenotypic characteristics were found in many organs [10–33]. Thus, it leads us to hypothesize that TCs may exist in all organs [11]. However, it is still unclear if TCs are present in cardiac valves. To our knowledge, this study shows, presumably for the first time, the existence of TCs in the interior of human cardiac valves, including mitral valve, tricuspid valve and aortic valve.

## Materials and methods

### Samples

Human heart valves including mitral valve, tricuspid valve and aortic valve were obtained from prospective multiorgan donors who did not have cardiovascular pathology in cases in which technical reasons prevented transplantation. Studies were conducted under an institutional review board-approved protocol. This study was approved by the ethical committees of the First Affiliated Hospital of Nanjing Medical University (Protocol Number: 2011-SRFA-075). The investigation conforms to the principles that are outlined in the Declaration of Helsinki regarding the use of human tissues.

### Transmission electron microscopy

Heart valves were cut into small pieces of 1 mm^3^ and were fixed with 5% glutaraldehyde in phosphate buffer (0.1 M, pH 7.4) at 4°C overnight. After washed in phosphate buffer for four times followed by post-fixation with 1% osmium tetroxide in 0.1 M phosphate buffer for 2 hrs at 4°C, these tissues were dehydrated through graded alcohol at a concentration of 50, 70, 90, 100% for 30 min. for each procedure and embedded in Epon 812. Semi-thin sections were cut at 1.5 μm on a Leica Ultracut R (Solms, Germany) and stained with toluidine blue and were histologically analysed by using light microscopy. Ultrathin sections were cut at 70 nm and contrasted with uranyl acetate and lead citrate and were examined by using a JEM-1010 electron microscope (JEOL, Tokyo, Japan). The density of TCs was expressed as the mean of the total number of TCs/total area.

### Immunofluorescent staining

Frozen sections were sliced into a thickness of 6 μM, and then were post-fixed with 4% paraformaldehyde dissolved in 0.1 M phosphate buffer (pH = 7.4) for at least 15 min. After washed with phosphate buffer for three times, sections were immersed in 10% goat serum for 1 hr. After that, sections were incubated overnight at 4°C with rat monoclonal anti-CD34 (ab8158; Abcam, Cambridge, UK) and rabbit monoclonal to Vimentin (ab92547; Abcam), both were with the dilution of 1:100 in phosphate buffer and permeabilized by 0.25% Triton X-100 at the same time. On the second day, the sections were incubated to goat anti-rabbit labelled with rhodamine secondary antibodies (sc-362262; Santa Cruz, Dallas, TX, USA) and goat anti-rat labelled with FITC (sc-2011; Santa Cruz) diluted 1:200 in phosphate buffer for 1 hr. The sections were stained with 4′,6-diamidino-2-phenylindole (DAPI; ProLong® Gold, Life technology, Carlsbad, CA, USA). Similar procedures were used in rabbit monoclonal to PDGF Receptor-beta (ab32570, 1:100; Abcam) and rabbit polyclonal anti-C-Kit (ab5506, 1:100; Abcam).

## Results

**Fig. 1:**
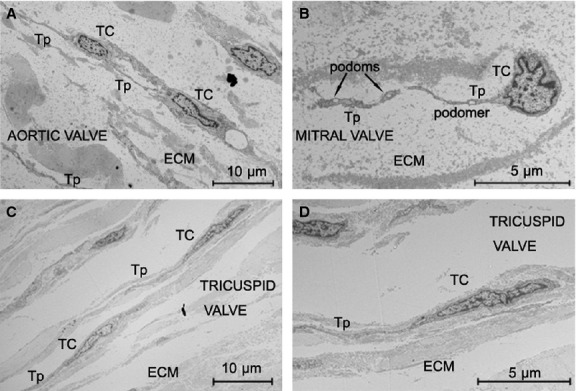
Electron microscope images show the existence of telocytes (TCs) in human heart valves. (**A**) TCs with telopodes (Tps) in the interstitial layer of aortic valve. (**B**) A TC with Tps in mitral valve. (**C**) TCs with Tps in tricuspid valve. Note the upper Tp which is more than 30 μm long. (**D**) A typical TC with Tps in tricuspid valve. ECM: extracellular matrix.

**Fig. 2:**
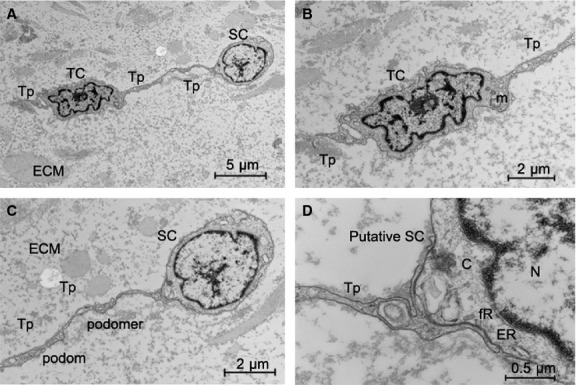
Electron microscope images show a telocyte (TC) in human mitral valve in close contact with a putative stem cell (SC). (**A**) A TC with telopodes (Tps) is feeding a SC. (**B**) Higher magnification of the TC with Tps. m: mitochondrion. (**C**) Higher magnification of Tps and a putative SC. (**D**) Higher magnification showing the junction between Tps and a putative SC. N: nucleus; c: centriole; ER: Endoplasmic reticulum; fR: free ribosomes; ECM: extracellular matrix.

Transmission electron microscopy has been shown to be the most precise way to identify TCs [5,6]. As shown in Figure 1, TCs were present in the interstitial layer of human cardiac valves in all three valve types. Figure 1A–D show TCs in the aortic valve, mitral valve and tricuspid valve, respectively. As observed in many other organs, TC had 2 or 3 long Tps emerged from the cell body [10,11]. Some of them have Tps more than 20 μm long. Telocytes are in close contacts (junctions) with putative stem cells [11,12,18,24,28]. Figure 2 shows a TC in human mitral valve with a complex junction established with a cell that has ultrastructural features of a stem (progenitor) cell, including an irregular shaped nucleus, large nucleolus, few endoplasmic reticulum cisternae and mitochondria, but numerous ribosomes [44].

**Fig. 3:**
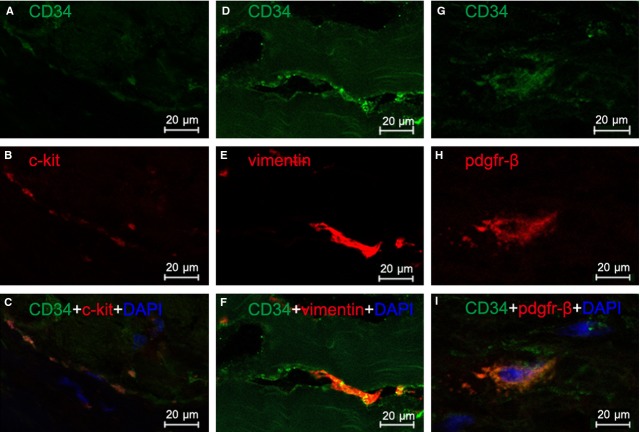
Human aortic valve: immunofluorescence labelling shows telocytes (TCs). Laser scanning confocol microscopy: CD34 and c-kit double immunofluorescence labelling showing (**A**) CD34 (green), (**B**) c-kit (red) and (**C**) co-localization (yellow) in a telocyte. CD34 and vimentin double immunofluorescence labelling showing (**D**) CD34 (green), (**E**) vimentin (red) and (**F**) co-localization (yellow) in a telocyte. CD34 and PDGF Receptor-β double immunofluorescence labelling showing (**G**) CD34 (green), (**H**) PDGFR-β (red) and (**I**) co-localization (yellow) in a telocyte. Nuclei are counterstained with DAPI (blue). Original magnification 400×; scale bar = 20 μm.

**Fig. 4:**
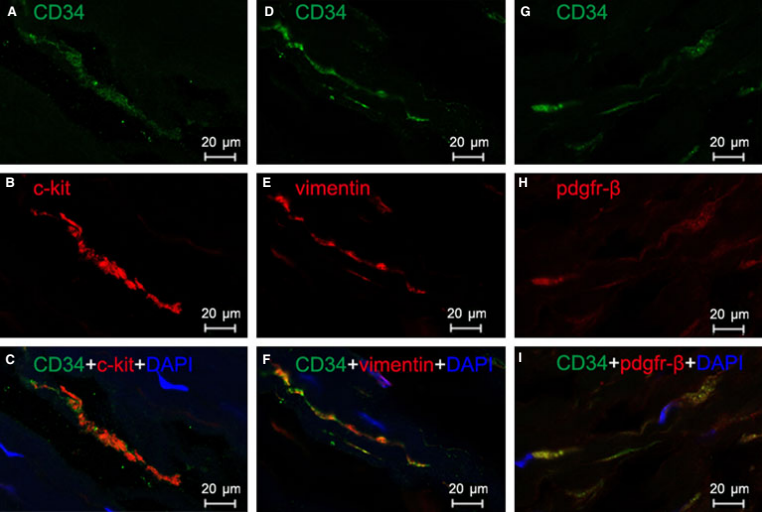
Human mitral valve: immunofluorescence labelling shows telocytes (TCs). Laser scanning confocol microscopy: CD34 and c-kit double immunofluorescence labelling showing (**A**) CD34 (green), (**B**) c-kit (red) and (**C**) co-localization (yellow) in a telocyte. CD34 and vimentin double immunofluorescence labelling showing (**D**) CD34 (green), (**E**) vimentin (red) and (**F**) co-localization (yellow) in a telocyte. CD34 and PDGF Receptor-β double immunofluorescence labelling showing (**G**) CD34 (green), (**H**) PDGFR-β (red) and (**I**) co-localization (yellow) in a telocyte. Nuclei are counterstained with DAPI (blue). Original magnification 400×; scale bar = 20 μm.

**Fig. 5:**
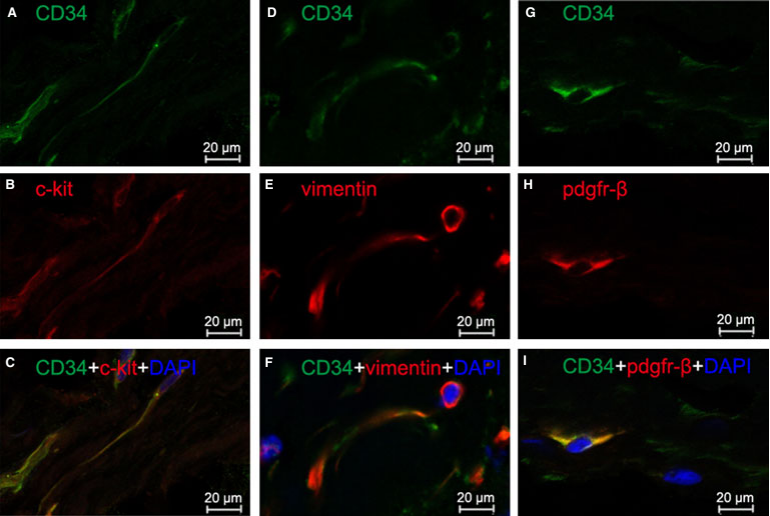
Human tricuspid valve: immunofluorescence labelling shows telocytes (TCs). Laser scanning confocol microscopy: CD34 and c-kit double immunofluorescence labelling showing (**A**) CD34 (green), (**B**) c-kit (red) and (**C**) co-localization (yellow) in a telocyte. CD34 and vimentin double immunofluorescence labelling showing (**D**) CD34 (green), (**E**) vimentin (red) and (**F**) co-localization (yellow) in a telocyte. CD34 and PDGF Receptor-β double immunofluorescence labelling showing (**G**) CD34 (green), (**H**) PDGFR-β (red) and (**I**) co-localization (yellow) in a telocyte. Nuclei are counterstained with DAPI (blue). Original magnification 400×; scale bar = 20 μm.

Three different double labelling immunofluorescence methods were used to present evidence for the presence of TCs in human heart valves. These were double labelling for CD34 and c-kit, CD34 and vimentin, and CD34 and PDGFR-β. Figure 3 shows double-positive cells in human aortic valve, whereas Figures 4 and 5 show double-positive cells in human mitral valve and tricuspid valve, respectively. Thus, three different double immune-staining methods consistently indicate the presence of TCs in human aortic, mitral and tricuspid valves. In addition, TCs can be found in both apex and base of heart valves, with a similar density of 27–28 cells/mm^2^ in mitral valve, tricuspid valve and aortic valve.

## Discussion

This study identified TCs as a novel distinct population of interstitial cells in human mitral, tricuspid and aortic valves by their typical ultrastructure and immunophenotype. The cellular body of TC is generally small and oval-shaped [11]. The striking ultrastructural feature of TCs is Tps [10,11]. Thus, TCs are different from the ‘traditional’ VICs. In addition, immunophenotypes of double positive for CD34 and c-kit, CD34 and vimentin, and CD34 and PDGFR-β also make TCs completely different from typical VICs [11].

This study adds novel evidence for the existence of TCs in human. However, the precise function of TCs in human valves remains to be established. It would be interesting to test the function of TCs *in vitro*. According to the literature, several relevant and potential roles could be proposed. The existence of telopodes and the 3D network indicates that TCs may function in the intercellular communication and regulation [10,11], either by sending signalling molecules *via* ectosomes [19,45] or by direct cell to cell contact [10]. Moreover, TCs might be essential as ‘nurse’ cells for progenitors [11].

Thus, TCs could represent novel target cells for the treatment of cardiovascular diseases related to the dysfunction of heart valves. The existence of TCs in heart valves provides a novel tool for understanding the essential biological process and even the pathological conditions that have not been determined in heart valve biology.

In conclusion, TCs are present in human heart valves. Telocytes may form a 3D network inside the cardiac valves, contributing to mechanical support (force distribution) and flexibility. We speculate that adding TCs to artificial heart valve may lead to better functional properties. It remains to be determined how TCs contributes to the ability of valve to re-establish normal structure and function following injury.
